# Haplotype-Resolved Chromatin Conformation Data Reveals Relationship Between Transposable Elements and Chromosomal Pairing

**DOI:** 10.1093/gbe/evaf222

**Published:** 2025-11-26

**Authors:** Luke K Genutis, Patrick Villanueva, Rita M Graze, Jumana AlHaj Abed, Lauren M McIntyre, Sergey V Nuzhdin

**Affiliations:** Molecular and Computational Biology Section, University of Southern California, Los Angeles, CA 90046, USA; Molecular and Computational Biology Section, University of Southern California, Los Angeles, CA 90046, USA; Department of Biological Sciences, Auburn University, Auburn, AL 36849, USA; Department of Genetics, Harvard Medical School, Boston, MA 02115, USA; Genetics Institute and Department of Molecular Genetics and Microbiology, University of Florida, Gainesville, FL 32603, USA; Molecular and Computational Biology Section, University of Southern California, Los Angeles, CA 90046, USA

**Keywords:** transposable element, *Drosophila* melanogaster, *Mus musculus*, chromosome pairing, Hi-C

## Abstract

Chromosomal structural changes happen when genomic stability is compromised, such as in disease or in species hybrids. In these contexts, diminished control of repetitive elements has been reported, but the reasons for this are not yet well understood. There are causal associations between repetitive elements and phenotypes such as disease progression, leading us to the hypothesis that chromosomal structure may be affected by transposable elements (TEs). In an intraspecific hybrid *Drosophila melanogaster* cell line (PnM), the degree of pairing among trans homologous chromosomes was affected by the presence of nearby TEs, in particular, LINE and LTR elements, such as Baggins1 or Gypsy. Chromosomal pairing was significantly lower in windows containing TEs than in windows without any TEs. Pairing was also affected by TEs in mouse, which suggests a possible general association between TEs and pairing that is highly conserved.

Significance Chromosomal structure is known to influence whether genes are able to be expressed. Understanding what factors are involved with the structure of chromosomes is important for studying other processes that might depend on this structure, such as how traits of interest are expressed or what it means for certain diseases in which this structure is disturbed. We have used data from a *Drosophila melanogaster* dataset to show that there is an association between stretches of repeated DNA sequence known as transposable elements (TEs) and chromosomal pairing. We have also used a *Mus musculus* dataset to validate this association in another distantly related species indicating that this process might be highly conserved among animals. This result helps to implicate the presence of nearby TEs and their potential role in this chromosomal pairing.

## Introduction

Chromosome structure is important for the maintenance of genetic fidelity during replication, and defects in processes related to chromosome structure are often highly deleterious and are observed in many diseases (Mirabella et al. 2016; Mirny et al. 2019). Heterochromatic regions can be transcriptionally silent (Richards and Elgin 2002), while open euchromatic regions are required for transcription to take place (Morrison and Thakur 2021). In mammals, heterochromatic regions contain many transposons, such as the class I retrotransposons that utilize an RNA intermediary: long interspersed nuclear elements (LINEs) and long terminal repeat (LTR) elements. Euchromatic regions often contain interspersed nuclear element (SINEs) repeats (Solovei et al. 2016). Class II transposons refer to DNA transposons that utilize a cut and paste mechanism to transpose throughout the genome; this class usually represents a far smaller share of the transposable element (TE) load among species, roughly 2.3% in *Drosophila melanogaster* (dmel) and 3% in humans (Mérel et al. 2020; Liang et al. 2024). Due to their deleterious effects when active and permitted to transpose to other positions in the genome, there is strong pressure and often redundancy between multiple silencing mechanisms to control TE activation, such as DNA methylation or Histone 3 Lysine 9 methylation (H3K9me) (Padeken et al. 2022) or through using PIWI RNA mediated silencing as in the germline in animals, including mammals and *Drosophila* (Senti and Brennecke 2010; Fablet et al. 2014; Romero-Soriano et al. 2017).

TEs can be relatively common in the genome, such as in the case of humans where half of the genomic content is inactive mobile DNA elements (Lander et al. 2001). In plants, TE content can vary dramatically, such as from 3% of the genome in *Utricularia gibba* (Ibarra-Laclette et al. 2013), to *Zea mays* where TEs account for almost 85% of the genome (Schnable et al. 2009). In some mammals, such as in mice and in humans, TEs can be found in the intronic regions of almost 90% of genes (Platt et al. 2018). In *Saccharomyces cerevisiae* (yeast), TEs are all LTR retrotransposon elements in five major classes, Ty1 to Ty5, and location and content was found to vary substantially depending on strain sampled (Bleykasten-Grosshans and Neuvéglise 2011; Bleykasten-Grosshans et al. 2013). Unlike in animals, RNAi activity and therefore RNAi-based methods to silence TE expression as in other species is lost in yeast (Drinnenberg et al. 2009). In addition, yeast lack 5-methyl-cytosine methylation activity that other species utilized to silence TEs in the genome (Bewick et al. 2019). Instead, yeast makes use of a dosage dependent mechanism to maintain control of TE copy number, by way of a Ty-derived capsid gene that inhibits a certain degree of transposition activity (Ahn et al. 2017).

These TEs can affect downstream gene expression changes, as well as the structure of chromatin (Maheshwari and Barbash 2011). TEs often play a role in disease, such as in cancer in a process called onco-exaptation (Babaian and Mager 2016) where these elements can drive the expression of oncogenes (Jang et al. 2019) or disrupt the expression of tumor-suppressing genes, such as APC (Miki et al. 1992; Burns 2017). LINE-1 retrotransposon insertions have also been shown to result in hemophilia A (Kazazian et al. 1988). A minimal model for euchromatic and heterochromatic formation has been suggested that mechanistically involve certain TEs, owing to the high degree of affinity that similar sequences have for each other (Solovei et al. 2016). It has also been extensively demonstrated that TE sequences could recruit other proteins that go on to post transcriptionally modify histone tails to favor compact chromatin packaging and heterochromatin formation (Trono 2015; Lawson et al. 2023). The molecular mechanisms by which repeat elements affect transcription and chromosome positioning in the nucleus are still being actively investigated (Wells and Feschotte 2020; Lawson et al. 2023).

In recent years, patterns of chromosome structure have been studied using chromosome conformational capture techniques, which permit the estimation of three dimensional proximity in chromosome sequence. High-throughput chromosome conformation capture (Hi-C) offers the ability to estimate chromosomal interactions genome wide, and patterns observed in Hi-C contact frequency maps reflect euchromatic or heterochromatic regions, among other subdomains such as topologically associated domains (TADs) (Lieberman-Aiden et al. 2009; Dixon et al. 2012; Lajoie et al. 2015; Rowley and Corces 2018). These domains have shown to have regulatory implications, containing enhancers and other regulatory regions, and the borders of these regions have been characterized with the presence of certain housekeeping genes, protein binding sites, as well as repeat elements (Dixon et al. 2012; Jin et al. 2013; Symmons et al. 2014).

Chromosome pairing refers to a state where homologous chromosomes, either in cis or trans, are paired into some defined structure that can lead to specific cis or trans regulatory outcomes (Apte and Meller 2012; Joyce et al. 2016). dmel has also been a useful model to study chromosome pairing structure relationships, since chromosomes in somatic cells are paired throughout development, and can influence gene expression in a process called transvection, where enhancers and silencers can act in trans on the paired homologous chromosome (Lewis 1954; Duncan 2002). Transvected loci with nearby genes have been shown to share transcriptional machinery and coordinate expression between these genes (Lim et al. 2018). Recent work using an intraspecific hybrid dmel model has shown that the degree of trans pairing between these homologous chromosomes can vary, with tighter paired regions associated with active chromatin, binding of transcription factors, and gene expression (Joyce et al. 2016; AlHaj Abed et al. 2019; Erceg et al. 2019).

In mammals, such as *Mus musculus*, homolog pairing is thought to occur transiently in a developmental or repair related fashion, and disturbed pairing is thought to contribute to aberrant gene expression or disease (Apte and Meller 2012; Joyce et al. 2016; Burns 2017). For example, in human renal oncocytomas, chromosome 19 has been observed to be paired and affect transcriptional regulation within the paired region (Koeman et al. 2008). In mice, transvection of methylation status has also been observed between homologs (Rassoulzadegan et al. 2002). While trans pairing between parental homologs in mice may be a sparse interaction to detect, it remains unclear how much repeats might contribute to pairing in general among homologs (Erceg et al. 2019).

In this study, we leverage data from an intraspecific dmel model originated from a cross between the Drosophila Genetic Reference Panel (DGRP) strains 439 and 057 (AlHaj Abed et al. 2019; Erceg et al. 2019) where there is genomic, transcriptomic, and chromosomal pairing data available, as well as an intraspecific mouse model (Du et al. 2017) with available chromosomal pairing data, in order to evaluate the hypothesis that TEs affect localized chromosomal pairing. The degree of pairing between homologous chromosomes was compared to genomic features such as density of single-nucleotide polymorphisms, the presence of annotated genes, expressed transcripts, and detected TEs. We found that regions that harbor TEs have significantly looser pairing than regions without TEs, with different classes of TEs more strongly associated with loose pairing than others. This observation is mirrored in an intraspecific mouse cross of C57BL/6N and PWK/PhJ (Du et al. 2017). Together, this suggests a generalized relationship between the presence of TEs and localization of chromosome pairs in the nucleus.

## Results

### Pairing Score and Its Association With Genomic Features in a dmel Intraspecific Hybrid

**Fig. 1.**
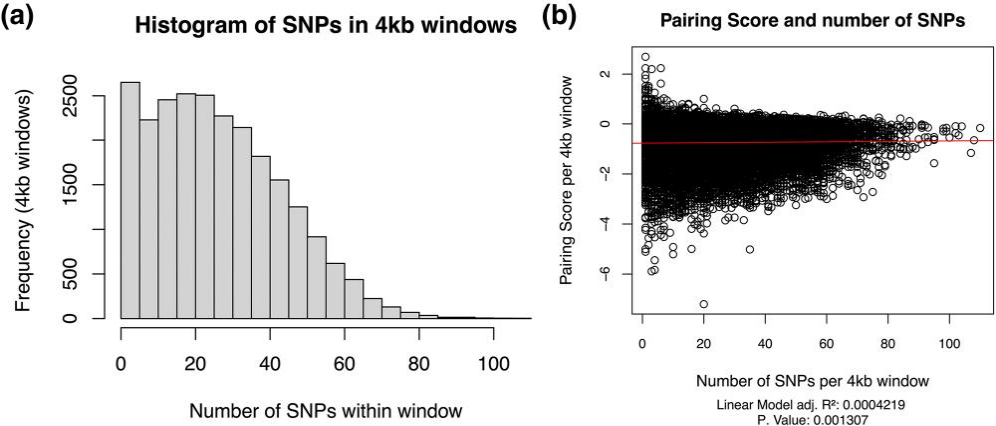
a) The distribution of SNPs is variable among 4 kb windows throughout the PnM hybrid cell line genome. Frequency refers to the number of 4 kb windows detected with the respective number of SNPs along the X axis. b) Pairing score over the trans-homolog Hi-C contact map is variable and does not appear to be associated with increased SNP density per 4 kb window.

We first sought to explore whether there was any relationship between pairing score, defined as the log_2_ average contact frequency for a given window in the genome from HI-C data, and the abundance of nearby SNPs in the intraspecific PnM (paternal DGRP-439 × maternal DGRP-057) dmel embryonic cell line data. We used available DNA sequencing (DNA-seq) data to call and quantify the number of SNPs detected within 4 kb windows among euchromatic regions defined in the dm3 genome of chromosomes 2 and 3, which is the resolution of the available pairing score data for this cell line. We find the frequency of SNPs to be approximately normally distributed around 20 SNPs per 4 kb window bin, although many 4 kb windows within the genome lack detected SNPs (Fig. 1a). To see if there was any relationship between the number of SNPs per 4 kb window and pairing, we then plotted the number of SNPs and the pairing score (Fig. 1b), finding no striking association between a high or low pairing score and increasing SNP counts within the 4 kb region.

**Fig. 2.**
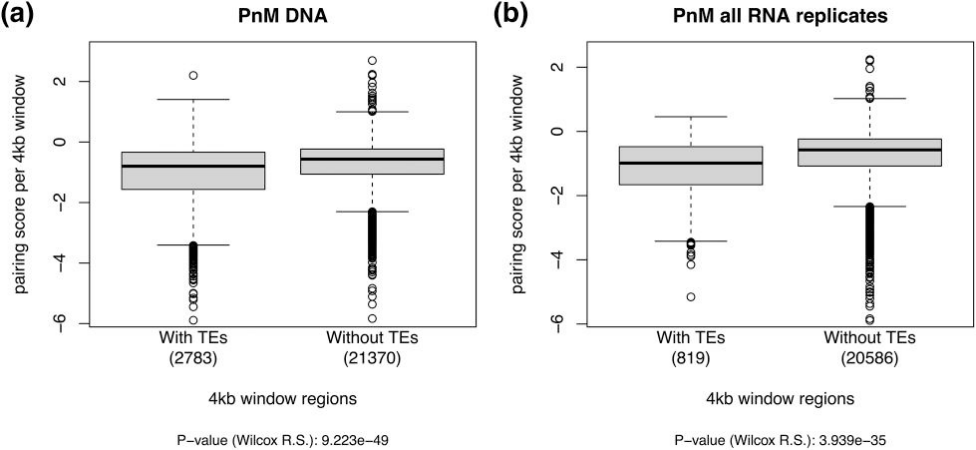
a) Pairing scores were compared between 4 kb windows containing genomic TEs and 4 kb windows without genomic TEs in the PnM cell line data. Pairing scores were significantly lower among these 4 kb window regions containing genomic TEs. b) Pairing scores were compared between 4 kb windows containing expressed TEs within all four PnM biological replicates and 4 kb windows without expressed TEs. Pairing scores were significantly lower among these 4 kb window regions containing expressed TEs in this replicate.

Since TEs could become dysregulated in hybrids (Romero-Soriano et al. 2017) and are often associated with neutral or deleterious effects on fitness (Mackay 1989; Mérel et al. 2020), we hypothesized that the presence of TEs affect pairing scores. We generated a list of TEs detected in the PnM DNA-seq data, TEs annotated in the reference dm3 genome, as well as TEs identified by TEfinder (Sohrab et al. 2021) in 4 kb windows. Using these same windows, we found that pairing scores are significantly lower (looser pairing) among windows containing TEs than windows without any TEs (Fig. 2a). Among the four PnM cell RNA sequencing (RNA-seq) replicates, the number of TEs expressed varied from 1597 to 2390, and 4 kb windows containing expressed TEs had significantly lower pairing than windows without any TEs (Fig. S1a to d). Windows that contained consistently expressed TEs across all replicates also had significantly lower pairing than regions without TEs (Fig. 2b).

**Fig. 3.**
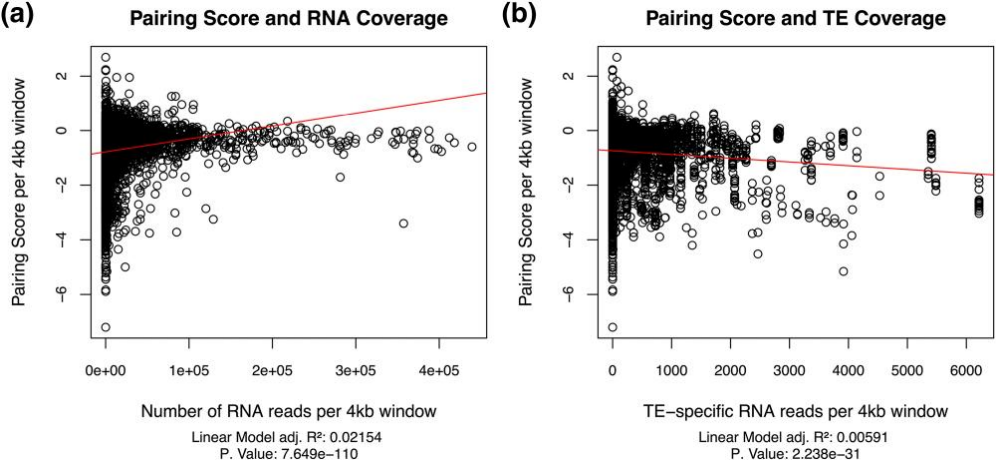
a) Pairing score and number of RNA reads per 4 kb window. There is a positive relationship between overall RNA expression and higher pairing scores. b) Pairing score and TE-specific RNA reads per 4 kb window. There is a significant negative relationship between TE expression and pairing scores.

Using RNA-seq data from four biological replicates, we also compared 4 kb windows and RNA coverage by measuring read depth per 4 kb window. We show a strong positive association with read depth and higher pairing score (Fig. 3a), in line with previous studies linking tighter pairing and gene expression (AlHaj Abed et al. 2019; Erceg et al. 2019). We also explored coverage of TE-specific reads among 4 kb windows, showing a significant negative trend with decreasing pairing scores as TE read depth increases (Fig. 3b).

Heterozygosity was also explored among the detected TEs. Pairing scores among 4 kb windows containing either TEs detected in the maternal or paternal DNA (heterozygous TEs), TEs detected in the maternal but not the paternal DNA (mat het TEs), TEs detected in the paternal but not the maternal DNA (pat het TEs), and TEs detected in the DNA of both parents were all significantly lower than pairing scores for the regions without any TEs (Fig. S2). This suggests that heterozygosity or the parent of origin does not bias the observed effect between TEs and pairing score as a generalized phenomenon.

**Fig. 4.**
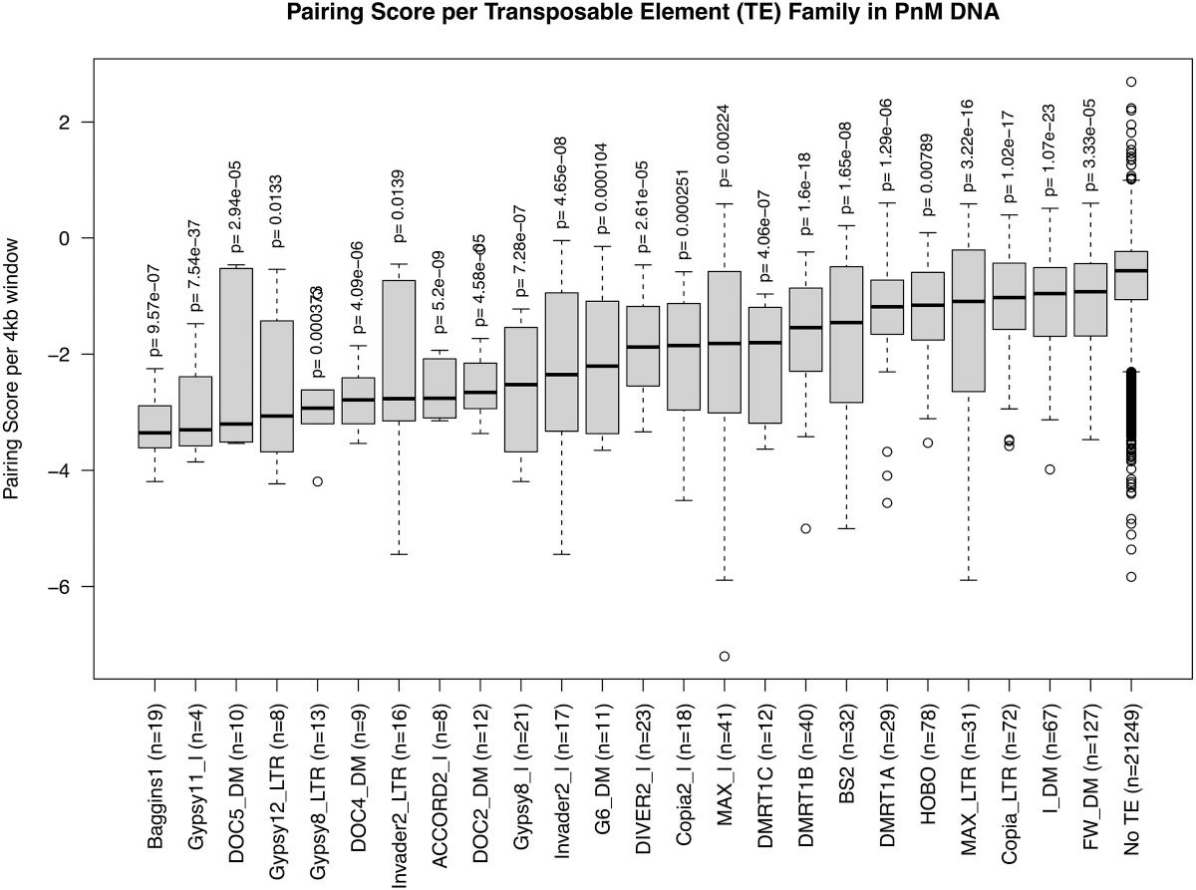
Pairing scores were calculated in 4 kb windows, and TEs were detected using TE finder within these 4 kb windows using the PnM DNA-seq data. For TE families detected in at least three 4 kb window regions, distributions of pairing scores were compared to windows not containing TEs (TEs) using a pairwise t test. Distributions significantly different (*P* < 0.05) are plotted.

The effect on pairing score was variable among different families of TEs, with some families showing a more significant association with lower pairing than others. Pairing scores were measured for each family of TE detected in at least three 4 kb windows (Fig. 4). Twenty-four TE families in total, including Baggins1, had median pairing scores lower than the windows not containing any TEs (No TE). Most of the TEs identified with significantly lower median pairing scores than No TE windows were among LINE or LTR class transposons (Table 1).

**Table 1:** Pairing scores were calculated in 4 kb windows, and TEs were detected using TE finder within these 4 kb windows using the PnM DNA-seq data

Transcript ID	*P* value	TE family	TE class
ACCORD2_I	9.57E-07	Gypsy	LTR
Baggins1	7.54E-37	LOA	LINE
BS2	2.94E-05	Jockey	LINE
Copia_LTR	1.33E-02	Copia	LTR
Copia2_I	3.73E-04	Copia	LTR
DIVER2_I	4.09E-06	Pao	LTR
DMRT1A	1.14E-02	R1	LINE
DMRT1B	5.20E-09	R1	LINE
DMRT1C	4.58E-05	R1	LINE
DOC2_DM	7.28E-07	Jockey	LINE
DOC4_DM	4.65E-08	Jockey	LINE
DOC5_DM	1.04E-04	Jockey	LINE
FW_DM	2.61E-05	Jockey	LINE
G6_DM	2.51E-04	Jockey	LINE
Gypsy11_I	2.24E-03	Gypsy	LTR
Gypsy12_LTR	4.06E-07	Gypsy	LTR
Gypsy8_I	1.60E-18	Gypsy	LTR
Gypsy8_LTR	1.65E-08	Gypsy	LTR
HOBO	1.29E-06	hAT	DNA
I_DM	7.89E-03	I	LINE
Invader2_I	3.22E-16	Gypsy	LTR
Invader2_LTR	1.02E-17	Gypsy	LTR
MAX_I	1.07E-23	Pao	LTR
MAX_LTR	3.33E-05	Pao	LTR

For TE families detected in at least three 4 kb window regions, distributions of pairing scores were compared to windows not containing TEs (no TE) using a pairwise t test. TEs that had a significantly lower pairing score than no TE regions are shown (*P* < 0.05, after Bonferroni correction).

### TE and Pairing Score Relationship in Other Species

In attempt to validate these initial findings in other systems, we used Hi-C data from a mouse line generated from an intraspecific cross. The contact frequencies for these Hi-C data are binned at 40 kb and mapped to both the maternal and paternal genomes, based on the presence of maternal or paternal-specific SNPs contained in either or both paired sequencing reads. While these are not necessarily purely trans reads as in the dmel derived PnM cell line data as they contain both cis pairs as well as pairs where one of the reads is unassigned to a particular parental genotype, these data would still indicate whether presence of TEs are affecting cis-chromosome interactions. This pairing interaction was calculated in a similar way as the PnM and intraspecific yeast data, using the log2 of the average contact frequency for each 40 kb bin across the genome. Samples were used from this dataset that were taken from the inner cell mass of preimplantation embryos, which is a stage of embryonic development where chromosomes have already begun to segregate and compartmentalize into an initial chromosomal structure (Du et al. 2017). The mean and median pairing scores were also calculated for each of the 810 annotated TEs in the mm9 mouse reference genome. These values were compared to the mean and median pairing scores for the entire genome of which there are contact frequencies by using a binomial test. The null hypothesis is that the distribution of TE groups within bins with calculated pairing scores lower than the overall genomic pairing score would be centered at 0.5, in other words, equal numbers of TEs with high and low pairing scores, indicative of no bias in pairing scores. Using both the maternal genome and paternal genome mapped datasets, we find that the mean pairing score distributions both were significantly greater than 0.5 (maternal, 509 windows with lower average pairing, estimate value = 0.63, *P* value = 2.61 × 10^−13^; paternal, 447 windows with lower average pairing, estimate value = 0.55, *P* value = 3.51 × 10^−3^; Table S1).

To identify which of these mouse TE families had significantly lower pairing scores than regions without any TEs, a Wilcoxon ranked sum test for the mean pairing score values of regions containing each TE family was used. Overall, we found seven TE families that were significant in both the maternal and paternally aligned data at the *P* < 0.05 level after correcting for multiple testing using the Bonferroni method (Table 2). These comprised of several LTR class repeat elements: RLTR14, RLTR144-int, RLTR33, RLTR40, and RMER17D2; the endogenous retrovirus MurERV4-int; and the satellite repeat ZP3AR. Several more LINE and LTR class elements were significant among the maternally aligned data (Table 2).

**Table 2:** Intraspecific hybrid mouse data were provided as aligned either to the maternal or paternal genome of the cross (see Du et al. [2017] for details)

Paternal genome		Maternal genome	
TE family	*P* value (corrected)	TE family	*P* value (corrected)
L1M4	4.16E-02	MLT1C	4.20E-02
L1MB5	3.78E-02	MurERV4-int	3.12E-02
L1MDb	8.61E-03	RLTR14	6.57E-03
LTRIS4	4.56E-02	RLTR14-int	5.86E-27
MurERV4-int	3.77E-02	RLTR33	1.83E-02
RLTR14	3.19E-02	RLTR40	3.28E-05
RLTR14-int	5.54E-27	RMER17D2	1.76E-03
RLTR33	2.66E-03	ZP3AR	9.92E-17
RLTR40	2.29E-05	…	…
RMER17D2	1.01E-03	…	…
RMER2	7.78E-03	…	…
ZP3AR	2.55E-12	…	…

These data were investigated using Wilcoxon ranked sum tests, and TEs that had a significantly lower pairing score than no TE regions are shown (*P* < 0.05, after Bonferroni correction).

We also looked to validate these findings in a more basal eukaryote for which haplotype resolved pairing score data were available, using a budding yeast dataset (*Saccharomyces cerevisiae*). In contrast to higher species, there are only five families of transposons, all class I retrotransposon elements, found in *Saccharomyces cerevisiae*: Ty1, Ty2, Ty4, and Ty5, belonging to the Ty1/Copia family, and Ty3 of the Ty3/Gypsy family (Bleykasten-Grosshans and Neuvéglise 2011). In this dataset, contact frequencies were provided in 32 kb window regions, amounting to 374 regions genome wide. While pairing scores were calculated for each TE family, because of the distributions of these TEs through the yeast genome and the 32 kb resolution of the Hi-C data, all window regions contained some TE content. We did not find a significant relationship between TE content and pairing scores, perhaps owing to the coarser 32 kb resolution of this dataset which precluded identification of windows that lack any TE content (Table S2 and S3).

## Discussion

Understanding the conformation of haplotypes in Hi-C data can reveal novel findings on chromosomal structures and function (AlHaj Abed et al. 2019; Erceg et al. 2019), and recently more methods have been developed to call haplotype resolved Hi-C results from diploid data (Tourdot et al. 2021; Hirata et al. 2022). Using haplotype resolved Hi-C data from an intraspecific hybrid model (AlHaj Abed et al. 2019; Erceg et al. 2019), we have evaluated the association between chromosomal pairing and various genomic features, such as the presence of nearby SNPs which was shown not to influence pairing (Fig. 1), as well as an association between decreasing pairing score and presence of TEs (Fig. 2). Since the analysis were limited to euchromatic regions defined in the dm3 genome, chromosome compaction state were controlled for. This association was more strongly associated with the presence of certain TE families relative to others (Fig. 4, Table 1 to 2).

Our observed lack of an effect toward pairing from SNPs agrees with previous findings in the literature regarding pairing. Using a hybrid yeast, it was shown that homologous chromosomes still preferentially interact with each other despite very high levels of divergence among parental species, and this was thought to reflect the greater role that DNA bound proteins play in reshaping chromosome architecture (Kim et al. 2017). Intriguingly our intraspecific mouse data suggest that maternally derived read pairs show looser pairing interactions among chromosome regions containing TEs than paternal regions that containing TEs (binomial test; 0.63 vs 0.55, respectively). Previous work on these data has shown that over the course of embryonic development, maternal and paternal genomes show differential segregation especially at the earliest zygotic stages, with maternal alleles more likely to interact across chromosomal compartments A and B (Du et al. 2017). This result, of the maternal genome having tighter pairing interactions and less segregation into discrete domains in the nucleus, suggests that they may be more likely to see disturbance in pairing scores in regions with TEs than for the paternal genome in this cross. Since there is more segregation and looser pairing interactions for the paternal genome, these chromosomes might be therefore less affected by having a TE in a nearby region since they were less paired to begin with relative to the maternally derived chromosomes. There has also been some evidence that mitochondrial inheritance can influence TE regulation, where swapping the maternal and paternal pairs of a cross can influence the rate of transposition by an order of magnitude in yeast hybrids (*S. cerevisiae × S. uvarum*) (Smukowski Heil et al. 2021).

Certain TEs are known to correlate with chromosomal interactions, SINE-family Alu which contains many binding sites for transcription factors (Polak and Domany 2006; Gu et al. 2016). SINE elements were found to have a higher proportion of contact frequency at TAD borders and are often found in euchromatic regions, while LINE and LTR were found to have a lower proportion at the TAD borders and are more typically found in heterochromatic regions (Solovei et al. 2016; Li et al. 2022). Among our results for the intraspecific PnM dmel data, most of these TEs associated with lower pairing score were LINE or LTR elements (Table 1). We show that certain elements, such as Baggins1, a LINE element, were shown to be significantly associated with lower pairing (Fig. 3, Table 1). Gypsy, an LTR class retrotransposon, is one of the most abundant TEs by copy number (Mérel et al. 2020), and we detect several Gypsy family TEs that were significantly associated with a lower median pairing score than regions without TEs (Fig. 3, Table 1 to 2). In consideration of the intraspecific mouse hybrid dataset, we also found seven TE families that were significant between the data aligned to either parental genomes, the majority of which being either LINE or LTR class retroelements, in concordance with the enriched classes observed among the dmel data (Table 2).

There are certain technological limitations inherent to our approach in annotating TEs. We chose a conservative approach using the most data available for each dataset studied. For the PnM dmel data, we were able to use TEfinder (Sohrab et al. 2021) to call TEs annotated in the reference genome as well as novel insertions, which requires a read to have some overlap between TE and mappable unique sequence, and unmappable reads containing only TE content are therefore discarded. For the mouse and yeast data, only Hi-C contract frequency matrices were available, so these were used along with reference genome TE annotation tracks to explore the relationship between pairing score and TE positions. This approach has several shortcomings such as the fact that actual TE content may vary among strains within a given species (Bleykasten-Grosshans et al. 2013) and the reliance on short read data which limits analysis to reads that can be technically mapped to some unique sequence (Sohrab et al. 2021). Long-read sequencing, on the other hand, offers read lengths sufficient to span the entire length of most TEs in the genome (Mohamed et al. 2020). Future work on the relationship between TEs and chromosomal pairing is therefore recommended to use a multiomic approach for a given dataset, where Hi-C data along with long-read sequencing data are both collected.

Among the yeast data, we were not able to detect windows without TE content and therefore could not compare pairing scores between TE and no TE windows, due to the Hi-C bin resolution of 32 kb. Yeast TEs are thought to distribute on average an insertion every 25.2 kb to 39.4 kb depending on chromosome analyzed and strain used to generate TE calls (Kim et al. 1998). Therefore, it may not be wholly unexpected to find that most regions do contain TEs at a 32 kb HI-C bin resolution in yeast.

Overall, we have developed an approach to studying TE dynamics using Hi-C data and have used this to show a putative association between trans chromosomal pairing, or pairing score, and nearby TEs classes—certain families of which are very strongly associated with lower pairing scores relative to others (Tables S2 and S3). These results provide insight into the role of TEs and chromosome structure, especially in contexts where control over TEs is disrupted, such as in hybrids or in diseases. Knowing the class of TEs associated with specific chromosomal structures could make these elements and their vicinity good candidate regions for attenuating chromosome structural dynamics.

## Materials and Methods

### Data Acquisition

RNA-seq data from four biological replicates of the PnM hybrid cell line were downloaded from the gene expression omnibus (GSE121256) using SRA toolkit (v. 2.9.6; SRA toolkit development team https://github.com/ncbi/sra–tools). DNA-seq data for the PnM hybrid cell line and the paternal (DGRP-439) and the maternal (DGRP-057) strains of dmel used to generate the PnM hybrid embryos, as well as a genomic track of pairing and cis scores in 4 kb genomic windows across chromosomes 2L, 2R, 3L, and 3R, all euchromatic regions, were generated as previously described from Hi-C contact matrices available on HiGlass (Kerpedjiev et al. 2018; AlHaj Abed et al. 2019; Erceg et al. 2019). Our downstream genomic analyses are limited to these chromosomes where we have pairing score data available. Reference TE annotation for the dm3 genome was downloaded from the Hammell lab (https://hammelllab.labsites.cshl.edu/contact/).

Hi-C data from budding yeast *(Saccharomyces cerevisiae*), consisting of a matrix of contact frequencies and chromosomal coordinates for a cross of DBVPG6044 and Y12 strains (Kim et al. 2017), were downloaded from NCBI Gene Expression Omnibus (GEO) (GSE88952) using SRA toolkit. Contact frequencies and chromosomal coordinates representing Hi-C data from mouse preimplantation embryos generated from a cross of C57BL/6N and PWK/PhJ were also downloaded from GEO (GSE82185) (Du et al. 2017). Pregenerated indices of repeat elements for the mouse mm9 reference genome were downloaded from the Hammell lab (https://hammelllab.labsites.cshl.edu/contact/).

Custom R (v. 4.2.2; The R Foundation) functions were used to calculate pairing scores from these contact frequencies according to the methods described in AlHaj Abed et al. (2019) and Erceg et al. (2019). The full pipeline for bioinformatics analysis is available on github (https://github.com/genutis/ps_manuscript_repo).

### Sequencing Alignment and Variant Calling

RNA data for the PnM hybrid cell line were aligned to the dm3 reference genome using STAR (v. 2.7.0) (Dobin et al. 2013). DNA data were trimmed using seqtq trimfq (v. 1.3; https://github.com/lh3/seqtk) aligned using bwa mem (v. 0.7.17) (Li 2013) to the dm3 reference genome. Samtools (v. 1.10) (Li et al. 2009) was used to sort and remove PCR duplicates. Bcftools (v. 1.12) (Danecek et al. 2021) mpileup (−min-MQ 20 –min-BQ 20), call, norm, and filter (-i'INFO/DP > 80 & QUAL > 200′) were used to pile up alignments on the reference genome and to normalize and filter called sequence variants. Coverage was measured for aligned NGS data using bedtools (v.2.30.0) (Quinlan and Hall 2010) multicov in 4 kb windows to correspond with the resolution of the pairing and cis score data.

### Transposable Element Calling and Data Aggregation

TEfinder (Sohrab et al. 2021) was used to call TEs that were both novel and annotated in the reference genome in both the PnM DNA and RNA data and supported by at least three discordant reads at the junction of the TE and nonrepetitive sequence, and reads which are unable to be uniquely mapped are discarded from this analysis. These data were aggregated in 4 kb windows along with the calculated TE-specific sequencing coverage, dm3 reference genes within each 4 kb window, and the number of genomic variants per window using bedtools^44^ intersect and bedtools groupby, as well as custom bash scripts. Aggregated TE calls, sequencing information, and pairing scores were statistically analyzed and visualized using custom R scripts. For per family TE calling, only families detected in at least three 4 kb windows were considered for analysis, and only families significant at *P* < 0.05 were plotted (pairwise t test).

For data such as the budding yeast and mouse datasets in which only the HI-C contact frequencies were available, TE annotations from each species respective genomic reference sequences were used. These data and reference sequences containing TE coordinates were used to compare pairing scores and TE content using custom R scripts and the *GenomicRanges* R package (v. 3.16) (Lawrence et al. 2013). Wilcoxon ranked sum and binomial tests in R were used to calculate statistical significance. Bonferroni testing was used when applicable for multiple test corrections.

## Supplementary Material

evaf222_Supplementary_Data

## Data Availability

The data analyzed in this study are available at NCBI GEO under the following accession numbers: GSE121255, GSE121256, GSE88952, and GSE82185. Pairing score data are available on the project github repository (https://github.com/genutis/ps_manuscript_repo).
