# Supplementary material for: Haplotype-Resolved Chromatin Conformation Data Reveals Relationship Between Transposable Elements and Chromosomal Pairing
Source: Genome Biol Evol. 2025 Nov 26;17(12):evaf222. doi: 10.1093/gbe/evaf222 (PMC12696580; doi:10.1093/gbe/evaf222)
Supplement: evaf222_Supplementary_Data [file evaf222_supplementary_data.zip › hic_tes_gbe_lg_supplemental_figures_07_25_25.docx]

Supplementary Figure 1

A. B.

C. D.

**Supplementary Figure 1.** Pairing scores were compared between 4kb windows containing expressed TEs and 4kb windows without expressed TEs in the PnM cell line data for the first (**A**), the second (**B**), the third (**C**), or the fourth (**D**) biological replicate with sequenced RNA. Pairing scores were significantly lower among these 4kb window regions containing expressed TEs in all individual replicates.

Supplementary Figure 2

**Supplementary Figure 2.** Pairing scores were calculated in 4kb windows, and transposable elements (TEs) were detected using TE finder within these 4kb windows using the PnM DNA sequencing data. TEs were further grouped based on the TE call of either the Maternal or Paternal DNA sequence data. Heterozygous TEs contain windows where a TE was detected in either parent. Maternal Het. TEs contain windows where a TE was detected in the maternal DNA sequence. Paternal Het TEs contain windows where a TE was detected in the paternal DNA sequence. Homozygous TEs contain windows where there was a TE in both parent’s sequence. No TEs contains windows that did not have a TE in the PnM DNA data.

**Supplementary Figure 3.** Pairing scores were calculated in 4kb windows, and transposable elements (TEs) were detected using TE finder within these 4kb windows using the PnM DNA sequencing data. For each bin, it was also calculated how many base pairs within each 4kb window contained overlapping TE sequence, and this was plotted against the pairing score for each window.
